# Hierarchically guided in situ nanolaminography for the visualisation of damage nucleation in alloy sheets

**DOI:** 10.1038/s41598-022-27035-8

**Published:** 2023-01-19

**Authors:** Mathias Hurst, Lukas Helfen, Thilo F. Morgeneyer, Heikki Suhonen, Ante Buljac, François Hild, Jussi-Petteri Suuronen, Tilo Baumbach, Daniel Hänschke

**Affiliations:** 1 Laboratory for Applications of Synchrotron Radiation, Karlsruhe Institute of Technology, 76131 Karlsruhe, Germany; 2 Institute for Photon Science and Synchrotron Radiation, Karlsruhe Institute of Technology, 76344 Eggenstein-Leopoldshafen, Germany; 3 Institut Laue–Langevin, 38042 Grenoble, France; 4 Centre des Matériaux, Mines Paris, PSL University, 91003 Evry, France; 5 Department of Physics, University of Helsinki, 00560 Helsinki, Finland; 6 ESRF-The European Synchrotron, 38043 Grenoble, France; 7 Xploraytion GmbH, 10625 Berlin, Germany; 8 Université Paris-Saclay, CentraleSupélec, ENS Paris-Saclay, CNRS, LMPS, 91190 Gif-sur-Yvette, France

**Keywords:** Engineering, Materials science, Nanoscience and technology, Optics and photonics, Physics

## Abstract

Hierarchical guidance is developed for three-dimensional (3D) nanoscale X-ray imaging, enabling identification, refinement, and tracking of regions of interest (ROIs) within specimens considerably exceeding the field of view. This opens up new possibilities for in situ investigations. Experimentally, the approach takes advantage of rapid multiscale measurements based on magnified projection microscopy featuring continuous zoom capabilities. Immediate and continuous feedback on the subsequent experimental progress is enabled by suitable on-the-fly data processing. For this, by theoretical justification and experimental validation, so-called quasi-particle phase-retrieval is generalised to conical-beam conditions, being key for sufficiently fast computation without significant loss of imaging quality and resolution compared to common approaches for holographic microscopy. Exploiting 3D laminography, particularly suited for imaging of ROIs in laterally extended plate-like samples, the potential of hierarchical guidance is demonstrated by the in situ investigation of damage nucleation inside alloy sheets under engineering-relevant boundary conditions, providing novel insight into the nanoscale morphological development of void and particle clusters under mechanical load. Combined with digital volume correlation, we study deformation kinematics with unprecedented spatial resolution. Correlation of mesoscale (i.e. strain fields) and nanoscale (i.e. particle cracking) evolution opens new routes for the understanding of damage nucleation within sheet materials with application-relevant dimensions.

## Introduction

Modern X-ray microscopy allows the non-destructive three-dimensional (3D) investigation of samples at nanoscale resolution^1^ and its potential for the study of in situ processes has already been demonstrated^2^. But often the possibilities for sample miniaturisation are limited, e.g. by the need for preserved boundary conditions or the danger of local disintegration by sample extraction. They also cannot keep up with the correspondingly reducing field of view (FOV) related to the increasing spatial resolution enabled by the progress of X-ray microscopy. For this reason, local 3D imaging approaches like local computed tomography (CT)^1^ or computed laminography (CL)^3^ play an increasingly important role for many applications related. For these local techniques, however, the identification and selection of possible regions of interest (ROIs) within the much larger samples is often hindered or completely precluded by the excessive superposition of features in the detected two-dimensional (2D) projection images. This is further complicated for in situ studies, where samples may undergo additional morphological changes or displacements. In consequence, new measurement strategies and techniques are required for the identification and continuous readjustment of the imaged sample sub-volume containing the features of interest. In this context, a serious complication arises from the formation of Fresnel diffraction patterns in the recorded images, caused by the propagation of the X-ray wave front from the sample to the detection plane. This in particular applies to magnified projection microscopy where the inherent conical beam effectively enhances the impact of Fresnel diffraction on the detected image. Consequently, in most cases a direct interpretation of the measured raw data as well as of 3D reconstructions based directly on raw data is not possible without prior image processing by means of suitable phase-retrieval algorithms.

**Figure 1:**
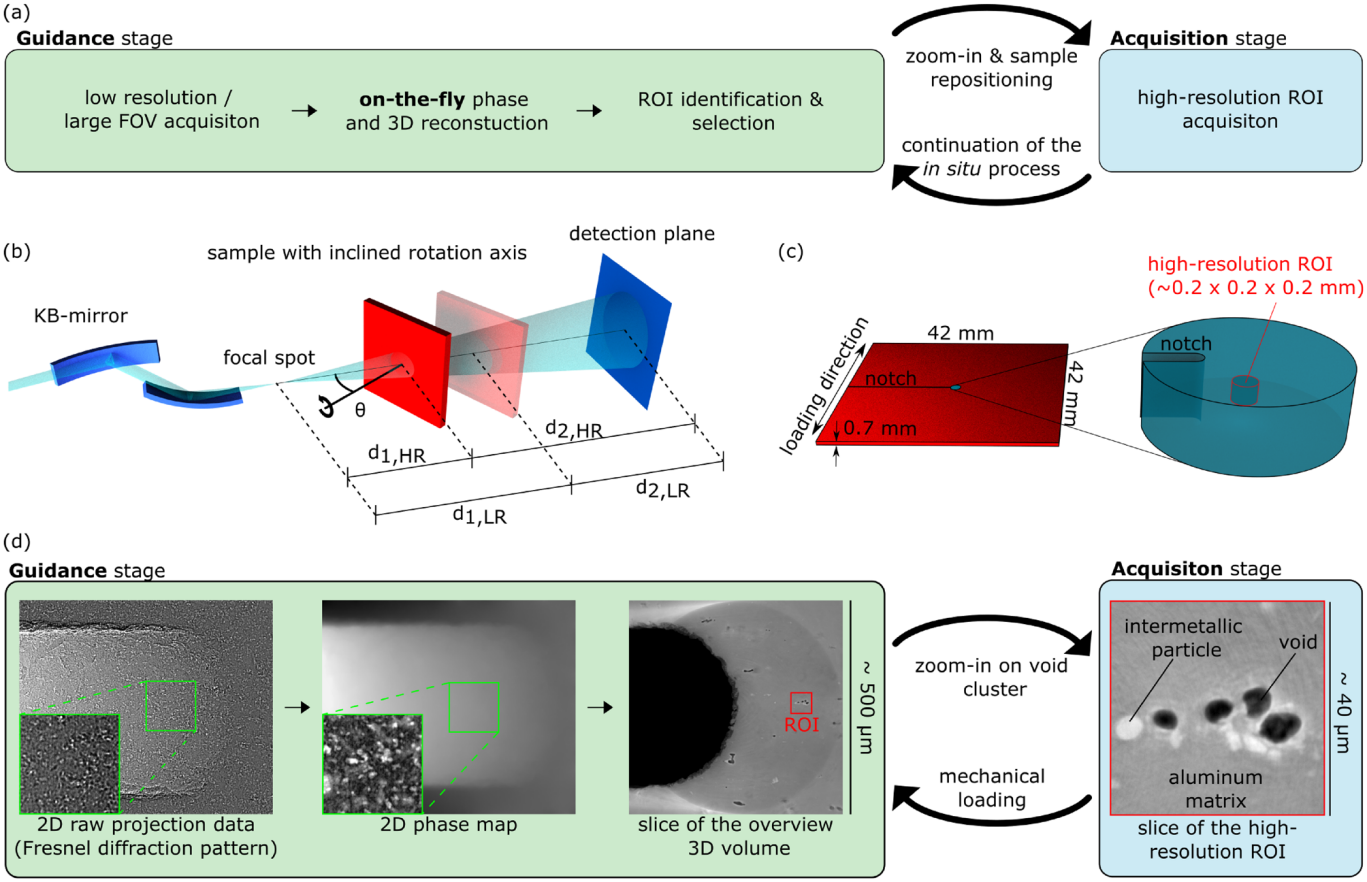
(**a**) Principle of the proposed hierarchical guidance. (**b**) KB-mirror-based magnified projection microscopy with zoom capability, combined with a laminographic scan geometry with tilt angle $\theta$. (**c**) Illustration of location and size of the high-resolution ROI, as shown in (**d**), within the macroscopic sample investigated in situ during loading under application-relevant boundary conditions. (**d**) Workflow of hierarchical guidance introduced in (**a**) applied for the in situ study of nanoscale processes within the sample of (**c**) during ductile fracture. Only after on-the-fly phase-retrieval and subsequent 3D laminographic reconstruction the identification and tracking of relevant void clusters (red rectangle) is possible, e.g for ROI identification and adjustment during the measurement. Before, in the corresponding regions of the raw and phase retrieved projection images (marked in green), the ROI features are indiscernible due to Fresnel diffraction and strong superposition of the faint contrasts associated. For visualisation purposes, here the contrast of the zoomed phase map has been enhanced by high-pass filtering.

We here introduce so-called hierarchical guidance for 3D X-ray microscopy, overcoming the above limitations of 3D nano-imaging by enabling the identification and refinement of the inherently small ROIs inside considerably larger samples. Particularly for in situ imaging experiments aiming on such high spatial resolution, accurate ROI tracking is considered a prerequisite. In short, as illustrated in Fig. 1a, the approach is based on the acquisition of multiscale data sets, directly followed by (ideally even simultaneous) suitable on-the-fly 2D phase and 3D volume reconstruction. The obtained immediate hierarchical 3D picture of the current sample state enables a direct feedback for the subsequent data acquisition, in particular for a continuous adjustment of dynamically evolving ROIs and their high-resolution 3D visualisation.

The required zoom capability down to the nanoscale is realised by means of projection microscopy^4^, which allows for a flexible adjustment of the magnification and related FOV by translating the sample between focal spot and detection plane^1,5^, see Fig. 1b. A sufficiently precise sample rotation stage equipped with motorised actuators for lateral sample translations enables 3D tomographic imaging with adequate ROI adjustment. In order to investigate flat and laterally extended sample geometries, we implement so-called computed laminography (CL), which is particularly suited for the screening of large sample areas and the zoom-in on local high-resolution ROIs^3,6,7^. For the realisation of the crucial on-the-fly (i.e. sufficiently fast) phase-retrieval suited for high-fidelity and high-resolution imaging (i.e. accounting for the typical Fresnel diffraction patterns in the holographic regime), we extend the so-called quasi-particle (QP) approach^8,9^ to a conical beam. For this, we carefully justify this extension theoretically, followed by a validation by simulations as well as by application to experimental data. In particular, we show that the obtained phase reconstruction of the magnified holographic data (i.e. with relatively small Fresnel numbers) does not suffer significantly regarding image quality compared to established multi-distance^1,5^ and/or iterative^2,10^ phase-retrieval approaches, all of which are considerably more time-consuming regarding measurement and/or phase-retrieval computation, respectively, and therefore not well applicable here. A similar on-the-fly capability like the extended non-iterative single-distance QP phase-retrieval is currently only achieved for data acquired in the edge-enhancement regime^11–13^, but which does not provide sufficient spatial resolution for our purposes. After phase-retrieval, the obtained phase images are directly streamed to a state-of-the-art GPU-based 3D tomographic or laminographic reconstruction pipeline^12^, resulting in a total data processing time sufficiently short for true on-the-fly execution. This means that directly at the end of the experimental data acquisition, or only with negligible delay, a picture of the internal sample morphology is accessible as overview or for browsing in detail, enabling immediate feedback for decision-making regarding subsequent experimental steps.

In order to demonstrate the potential of the proposed hierarchically guided X-ray nanolaminography, we apply this approach to enable in situ 3D imaging with deep sub-micrometre spatial resolution for studying damage nucleation during ductile fracture inside an aluminium alloy sheet (Al-Cu-Mg (AA2139T3)) under plastic deformation. Here only sub-micrometre spatial resolution allows observing the relevant void formation and secondary phase particle cracking mechanisms, while in the same time a macroscopic sample size is indispensable for the realisation of the strain state required, see Fig. 1c. Without a suitable guidance as provided by the presented approach, the identification and tracking of the relevant sample features turns out to be impossible, see Fig. 1d.

With increasing environmental and economical demand, recycling and circular industries become of interest for alloy manufacturers, in particular for aluminium alloys. However, recycling of alloys of different composition may result in an increase of impurity and associated intermetallic particle content that in turn may affect important mechanical properties like the formability^14^. Here new experimental insight and corresponding models are needed to be able to predict the effect of particle content on mechanical properties. Particle nature, size and distribution are also known to play an important role in this process^15^. For the development of such new models, the knowledge of the in situ microstructural development (including voids and secondary phase particles) as well as its correlation to the strain magnitude and conditions within the material is crucial. Previous studies on damage nucleation and growth have been performed in situ at the microscale^16,17^, but only ex situ at the nanoscale^18–20^. Here the study presented opens new possibilities for obtaining a comprehensive picture of damage processes in materials via the combination of 3D nanoscale resolution and in situ measurement. Thereby, hierarchical guidance is key for identifying, zooming-in-on and tracking damage-relevant clusters of voids and secondary phase particles in situ, with high resolution, and still within sufficiently large samples maintaining the application-relevant boundary conditions.

Moreover, ductile fracture^21^ plays a crucial role for technological applications of the investigated material and is typically split into three characteristic steps: void nucleation, void growth and void coalescence. Continuum damage models are known to describe and predict well the void growth and coalescence phase^22,23^, while usually only phenomenological models are available for the void nucleation phase. The latter depict the void nucleation rate as a Gaussian function having a maximum rate at a critical strain^24^. However, the contributions of stress and strain to the nucleation process are currently not sufficiently well known. Moreover, all these models cannot account for morphology, size, and nature of particles. We here show the feasibility of analysing nanoscale morphology and of measuring displacements *via* digital volume correlation (DVC) based on the obtained multiscale data, in this way enabling the correlation of mesoscale strained bands and nanoscale damage nucleation processes.

In the following, first the concept and principles of hierarchical guidance are introduced. For its implementation, the adaptation of QP phase-retrieval to the conical beam case plays a central role and is thus described in more detail. In particular a theoretical and experimental justification of its validity under the changed scaling conditions is given and the image quality is compared to established and commonly used phase-retrieval approaches for X-ray microscopy. In the second part, we demonstrate the application of the approach for the observation of an AA2139T3 aluminium alloy sheet during in situ mechanical testing. Here the main aspects of the experimental setup for the in situ loading test are described, followed by the nanoscale results obtained in combination with a DVC strain analysis.

## Methodical developments for hierarchically guided 3D nano-imaging

The experimental workflow of hierarchically guided X-ray microscopy is illustrated in Fig. 1a: first, a measurement with sufficiently low spatial resolution is performed, with its correspondingly large field of view aiming on a 3D overview picture of the sample investigated. By means of suitable on-the-fly data processing and visualisation, this low-resolution 3D map serves for guiding the selection and adjustment of the actual high-resolution ROI measurements, regarding location, suitable zoom factors, etc. Again, the same on-the-fly phase and 3D reconstruction procedure allows instantaneous and continuous quality assurance, including potential refinement of these high-resolution ROI measurements and the experimental parameters used. This is of particular interest for in situ studies, where the external control of process parameters may now react on the process history captured on the nanoscale, influencing e.g. the subsequently applied heating or mechanical loading, possibly followed by the next iteration of the scheme.

Instrumentally, as depicted in Fig. 1b, our implementation of the presented approach is based on a projection microscopy setup realised by means of a Kirkpatrick-Baez (KB) mirror system. The resulting zoom factor can be flexibly adjusted by positioning the sample along the optical axis^1^, its large energy bandwidth enables sub-second exposure times (here ${0.4}\,\text {s}$ per projection), and the achievable photon energy (here ${29.6}\,\text {ke}\text {V}$) allows penetrating also thick and optically dense samples. More details about the X-ray projection microscopy instrumentation and the performed measurements are given in the methods section. The 3D imaging capabilities are based on so-called X-ray computed laminography^3,6,7^, which generalises the conventional tomographic acquisition geometry by tilting the rotation axis with respect to the optical axis. In this way, high quality 3D imaging of laterally extended samples considerably exceeding the field of view becomes possible. In the setup used, the tilted laminographic rotation axis has a sphere of confusion below ${150}\,\text {nm}$, resulting in a similar achievable 3D spatial resolution. For ROI selection, a motorised translation system positions the sample relatively to this axis. Additional information about computed laminography is presented in the methods section.

Our data processing pipeline takes advantage of the highly efficient UFO framework^12^, complemented with the here presented single-step phase-retrieval algorithm, which is suited for microscopy in the holographic regime. In particular, the framework allows direct streaming of the raw data from the camera to GPUs where the data processing is performed on-the-fly i.e. phase-retrieval, tomographic filtering, and back-projection of the recorded Fresnel diffraction pattern is possible in parallel to the ongoing acquisition process. This enables a 3D sample visualisation immediately after data acquisition.

In Fig. 1d, a typical example of the experimental data recorded for the presented application of hierarchical guidance is illustrated, as well as its processing and the low-resolution and high-resolution 3D reconstructions obtained. Clearly, phase-retrieval and 3D reconstruction are crucial for the microscopic measurement in the holographic regime, since the features of interest can not be recognised in the raw and phase-retrieved projection images. Since both time-efficiency and high-resolution are crucial for the required phase-retrieval in the holographic regime (i.e. with relatively small Fresnel numbers), in the following a suitable extension of the QP phase-retrieval approach is carefully justified based on theoretical considerations, supported by a validation by simulations as well as by application to experimental data.

On the one hand, until now all published pipelines capable of on-the-fly phase-retrieval are limited to data recorded in the edge-enhancement regime^12,13^ (i.e. with relatively large Fresnel numbers as obtained by short propagation distances and typically for a parallel beam), which is a direct consequence of the employed single step phase-retrieval algorithm^11^. This algorithm is not applicable to holographic data obtained by X-ray projection microscopy. On the other hand, all published phase-retrieval approaches suited for projection microscopy require time-consuming multi-distance acquisitions^1,25^ or iterative procedures^10,26^, which allow compensating for non-measured frequencies and overcoming certain limitations of the underlying physical models. The multi-distance approaches^1,25^ require time-consuming measurements, the computation of several Fourier transforms and prior image registration. Depending on the number of iterations necessary^10,26,27^, the iterative approaches require the computation of ten to several thousands Fourier transforms, even with state-of-the art hardware resulting in typical reconstruction times of not less than ${2}\,\text {s}$ per projection^10^.

We here overcome these current speed limitations for high-quality phase-retrieval in the holographic regime by adapting the non-iterative, single-distance QP approach^8,9^ to a diverging cone-beam geometry. The resulting phase-retrieval technique requires the calculation of only a single frequency filter in Fourier space and is applicable to a broad range of samples, since the restrictions of so-called contrast-transfer-function based approaches^1,5^ concerning the samples optical properties are considerably relaxed. So far, however, the QP approach has been applied only to parallel beam conditions and thus the resulting imaging resolutions have been limited to about ${\sim 0.6}\,\upmu \text {m}$ (corresponding to the spatial resolution achieved by state-of-the-art X-ray detector systems^28^). By extending its application to cone-beam geometries the limitation in resolution is reduced to focal spot sizes of state-of-the-art focusing devices^29^. In the following, the cone-beam adapted version of the QP approach is built on the Fresnel scaling theorem^30^, but due to the strongly reduced Fresnel numbers the Fourier space coverage for single-distance acquisition changes drastically for a cone-beam compared to parallel beam conditions. Thus, we finally justify the application of the QP approach by a careful comparison to phase-retrieval approaches established for projection microscopy.

The QP approach^8^ is closely related to contrast-transfer-function (CTF) approaches^25,31^, which are based on certain assumptions on the optical properties of the sample under investigation (i.e. homogeneous or pure phase object). As a consequence, the QP approach involves a term which regularises frequencies that are corrupted by the violation of these assumptions^8^. The cone-beam adapted version of the QP approach results in the following relationship between the phase shift $\phi _{\text{sample}}$ introduced by the sample and the measured and subsequently empty beam corrected Fresnel diffraction pattern $I_{d_2}$:

1 $$\begin{aligned} \mathscr {F} \{ \phi _{\text{sample}} \} (\ {{\varvec{f}}}_{\bot , \textrm{eff}}) = \frac{\mathscr {F} \{ I_{d_2} - 1 \} (\ {{\varvec{f}}}_{\bot , \textrm{eff}})}{2\cdot \sin (\pi \lambda d_{\textrm{eff}} \left| \ {{\varvec{f}}}_{\bot , \textrm{eff}}\right| ^2) +10^{-\alpha }} \cdot A. \end{aligned}$$

In this expression, ${{\varvec{f}}}_{\bot , \text{eff}}$ denotes the transverse coordinate vector of frequency space, $d_{\textrm{eff}}$ the effective propagation distance, $\mathscr {F} \{ I_{d_2} \} (\ {{\varvec{f}}}_{\bot , \textrm{eff}})$ the Fourier transform of the intensity pattern in the detector plane, and $\alpha$ the parameter for regularisation of the zero frequencies. The relation of $I_{d_2}$, the raw Fresnel diffraction pattern $\tilde{I}_{d_2}$, and the detected beam profile without any sample $\tilde{I}^0_{d_2}$ are related by $I_{d_2}=\tilde{I}_{d_2}/\tilde{I}^0_{d_2}$, while the factor *A* is given by

2 $$\begin{aligned} A = {\left\{ \begin{array}{ll} 1 &{} |\pi \lambda d_{\textrm{eff}} \left| \ {{\varvec{f}}}_{\bot , \textrm{eff}}\right| ^2| \le \frac{\pi }{2}, \\ \Theta ( |\sin (\pi \lambda d_{\textrm{eff}} \left| \ {{\varvec{f}}}_{\bot , \textrm{eff}}\right| ^2)| - \varepsilon )&{} \textrm{elsewhere}. \end{array}\right. } \end{aligned}$$

**Figure 2:**
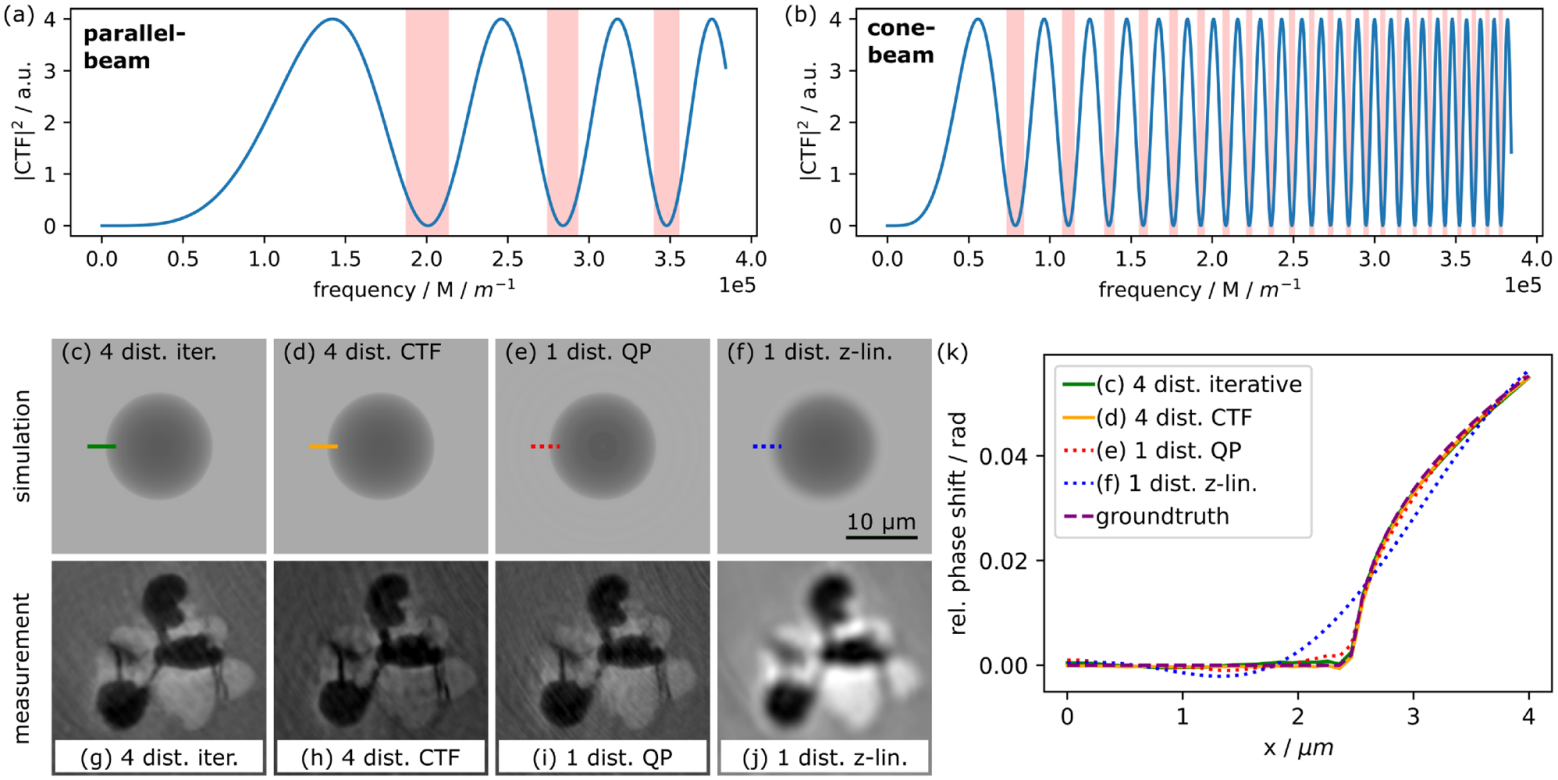
(**a**,**b**) Comparison of phase-contrast transfer for parallel- (Magnification $M=1$) and cone-beam configurations (magnification $M=6.5$) with parameters from experimental high-resolution scans, for the parallel beam case an effective pixel size of ${0.65}\,\upmu \text {m}$ is assumed. The red coloured frequencies are suppressed by the QP filter. The number and width of suppressed frequency bands changes drastically due to the cone-beam scaling. Images (**c**)–(**k**) compare different phase reconstruction methods for magnified projection microscopy. Simulated phase maps of a void-like inclusion are shown in sub-figures (**c**)–(**f**), measured laminographic slices of voids inside an AA2139T3 aluminium alloy are shown in sub-figure (**g**)–(**j**): (**c**,**g**) multi-distance approach with iterative refinement; (**d**, **h**) non-iterative multidistance CTF approach; (**e**,**i**) QP approach; (**f**,**j**) single-distance z-linearised approach. Sub-figure (**k**) shows the relative phase shift along the corresponding bars in (**c**)–(**f**).

Here, $\Theta$ denotes the Heaviside step function, while $\varepsilon$ is a parameter defining the width of the neglected frequency band around zero-crossings. Thus, the factor *A* allows suppressing frequencies where second order phase and attenuation contributions start to be dominant and thus corrupt the inversion of Fresnel propagation (i.e. influences of large phase shifts and absorption). As a result, this approach allows for the imaging of samples with optical properties not suitable for conventional CTF phase-retrieval due to considerably large phase variations, e.g. the here investigated ${0.7}\,\text {mm}$ thick Al-Cu-Mg (AA2139T3) aluminium alloy sheet with inhomogeneous inclusions. In order to enable QP-based cone-beam zooming, the presented filter is extended to a cone-beam configuration using an effective propagation distance known from the Fresnel scaling theorem^30,32,33^, i.e.

3 $$\begin{aligned} d_{\textrm{eff}} = \frac{d_{2}}{M}, \end{aligned}$$

with the geometrical magnification $M=\frac{d_1+d_2}{d_1}$ introduced by the cone-beam geometry. The propagation effects of a cone-beam with the real propagation distances $d_2$ correspond to the propagation effects in a parallel beam geometry with a much shorter propagation distance^1^ $d_{\text {eff}}$. In combination with the scaling of the frequency space coordinate ${{\varvec{f}}}_{\bot , \textrm{eff}}={{\varvec{f}}}_{\bot }\cdot M$ the argument of the sine function scales with factor *M*. Thus, in contrast to parallel beam propagation, already for relatively short propagation distances $d_2,$ many zero crossings are expected to occur in the power spectrum (see Fig. 2a,b). This phenomenon can be explained by the higher spatial frequencies now accessible due to the additional cone-beam magnification (smaller effective pixel size) and leads to a different Fourier space coverage compared to parallel-beam quasi-particle conditions: the number of minima is significantly increased, with a considerably narrower spacing in the power spectrum (see Fig. 2a,b).

In order to validate the above given properties of QP phase-retrieval for the now strongly modified Fourier space coverage, we compare its performance with that of the following three state-of-the-art phase imaging techniques: (i) multidistance acquisition taking into account all non-linearities (strong phase objects and strong absorption) of Fresnel propagation^26^, see Fig. 2c,g. The approach uses an initial phase map (e.g. retrieved based on Paganin’s approach^11^) as input for an iterative optimisation procedure between forward calculated, reconstructed phase maps and measured Fresnel diffraction patterns for several propagation distances (using 10 iterations). (ii) conventional multidistance CTF approach under the assumption of a homogeneous ($\delta \propto \beta$) and weak phase object^31^, see Fig. 2d,h. (iii) z-linearised single-distance approach similar to Paganin’s approach^11^, estimating the sine function by its argument, see Fig. 2f,j. Optimising Fourier space coverage, for the multidistance approaches $d_{2,i}\approx$ ${0.638}\,\text {m}$, ${0.637}\,\text {m}$, ${0.633}\,\text {m}$, ${0.623}\,\text {m}$ are assumed as propagation distances, while for the single-distance phase-retrieval $d_{2}\approx$ ${0.638}\,\text {m}$ has been used. The same distances have been used for simulated and experimental data. The simulated phase maps in Fig. 2c–f show a single void-like inclusion with maximum phase shifts up to ${\sim 0.1}\,\text {rad}$, however larger phase shifts can be treated by the QP approach as demonstrated by the experimental data or as shown in^8^.

Our comparison of the non-iterative CTF-based multidistance phase-retrieval (ii) in Fig. 2d,h and the QP approach in Fig. 2e,i reveals a superior image quality of the latter, despite the single distance acquisition. This difference can be explained by the optical properties of the sample, which are not in agreement with the assumptions of the native CTF approach, leading to low frequency, fringe like artefacts in the obtained images. Using multidistance acquisition followed by an iterative optimisation (i), the image quality is superior to the QP approach, even if the sample is not in agreement with the assumptions necessary for the CTF approach (Fig. 2c,g). For (iii), the approximation of the sine function by its argument is only valid for small frequencies, the suppression of high frequencies leads a loss of spatial resolution compared to QP. In particular, the intermediate frequencies are wrongly reconstructed, leading to the bright regions around objects visible in Fig. 2f,j. In Fig. 2k, the line plots indicated in Fig. 2c–f are compared, revealing clearly the inferior performance of (iii) under the here considered conditions, thus excluding it as alternative solution for the single-distance measurement scheme required for hierarchical guidance.

To summarise, the presented comparison of available phase-retrieval approaches shows that the cone-beam version of the QP approach is superior to the alternative single distance approach (iii) regarding the achievable resolution and image quality. The two alternative methods (i) and (ii), which are competitive in terms image quality are iterative and/or multidistance techniques, which are considerably more time consuming during measurement and/or reconstruction. The latter are thus not suited for fast or even on-the-fly computations. In contrast, the cone-beam version of QP turns out to be applicable for the projection data acquired by magnified projection microscopy^1,2^, providing both high resolution and on-the-fly capability as required for the hierarchically guiding approach introduced above.

## Nanoscale in situ investigation of damage nucleation in alloy sheets

In the following, the introduced hierarchical guidance plays a key role in the feasibility study of nanoscale in situ investigations of damage nucleation and growth in an AA2139 T3 aluminium alloy sheet during ductile fracture. The knowledge of these processes are highly relevant for understanding of the dependence of ductile fracture on the material nanostructure and strain conditions. This type of studies have so far been prevented by the lack of nanoscale in situ morphological data of such materials under mechanical loading.

**Figure 3:**
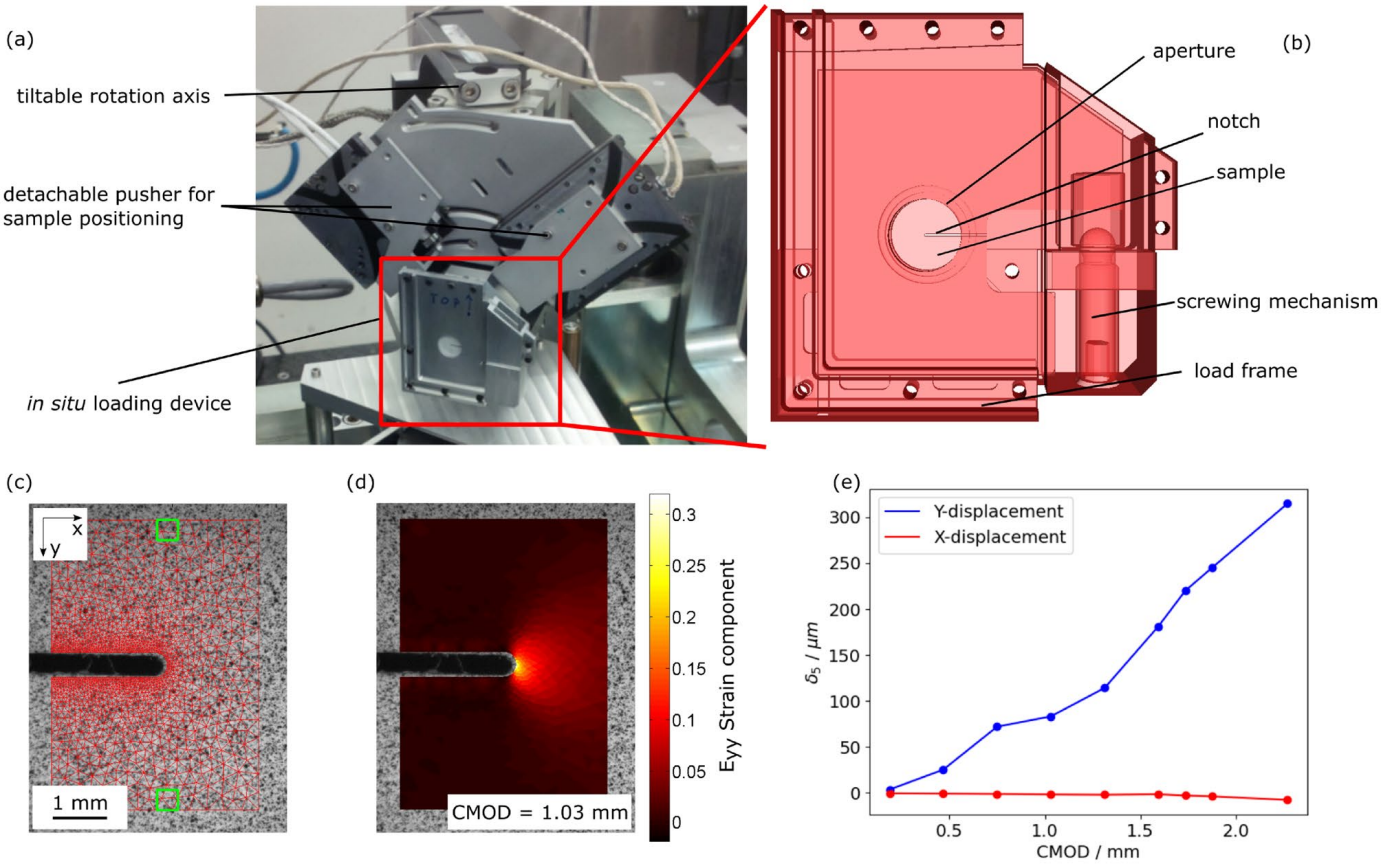
(**a**) Laminography setup for the presented nanoscale in situ measurements. (**b**) The used light-weight loading device including the notched sample. (**c**) Image of the superficial speckle pattern acquired with off-line visible light microscopy. (**d**) $E_{yy}$ component of the strain tensor at the surface of the sample measured by DIC. (**e**) Result of extensometer measurements based on the green squares in (**c**).

The nanolaminography measurements have been acquired with the setup shown in Fig. 3a and the details of the experimental imaging parameters can be found in the methods section. The loading device is attached with a magnetic system to the tilted rotation stage and is compatible with the dedicated pusher system for precise sample positioning. The small sphere of confusion of the setup of about ${150}\,\text {nm}$ allows for high-resolution CL at voxel sizes down to ${100}\,\text {nm}$. The in situ loading has been applied to a compact-tension-like shaped sample with dimensions of $42\, \times \, 42\, \times 0.7~\text {mm}^3$ (see Fig. 1b). This configuration creates a high stress triaxiality ($>1$) in the investigated ROI ahead of the notch root. For the loading of the sample a dedicated setup^34^ was used, shown in Fig. 3b. The loading rig is a specific light-weight construction, avoiding degradation of the rotation axis sphere of confusion. It consists of a load frame, a displacement controlled loading mechanism, and an aperture to minimise X-ray absorption. The loading state is quantified by the crack mouth opening displacement (CMOD), which is estimated from the screwing mechanism-based loading procedure. Despite the necessity of taking the loading rig off the rotation axis for mechanical loading and despite considerable sample deformation during the loading, hierarchical guidance allowed several ROIs to be tracked during the in situ measurement.

To measure the local displacements rather than $\text {CMOD}$ values, the sample surface is observed with an additional off-line optical microscope after each loading step. By correlating speckles painted to the sample surface, displacements can be measured *via* digital image correlation (DIC) (see Fig. 3c,d)^35^. The $E_{yy}$ strain component for an example loading step is shown in Fig. 3d. The green squares in Fig. 3c are used as a two point extensometer in order to determine the opening $\delta _5$ values with a measurement length of 5 mm^36^. The resulting local opening values are shown in Fig. 3e, revealing that the desired tensile loading in the y-direction is achieved by the device and sample geometry.

**Figure 4:**
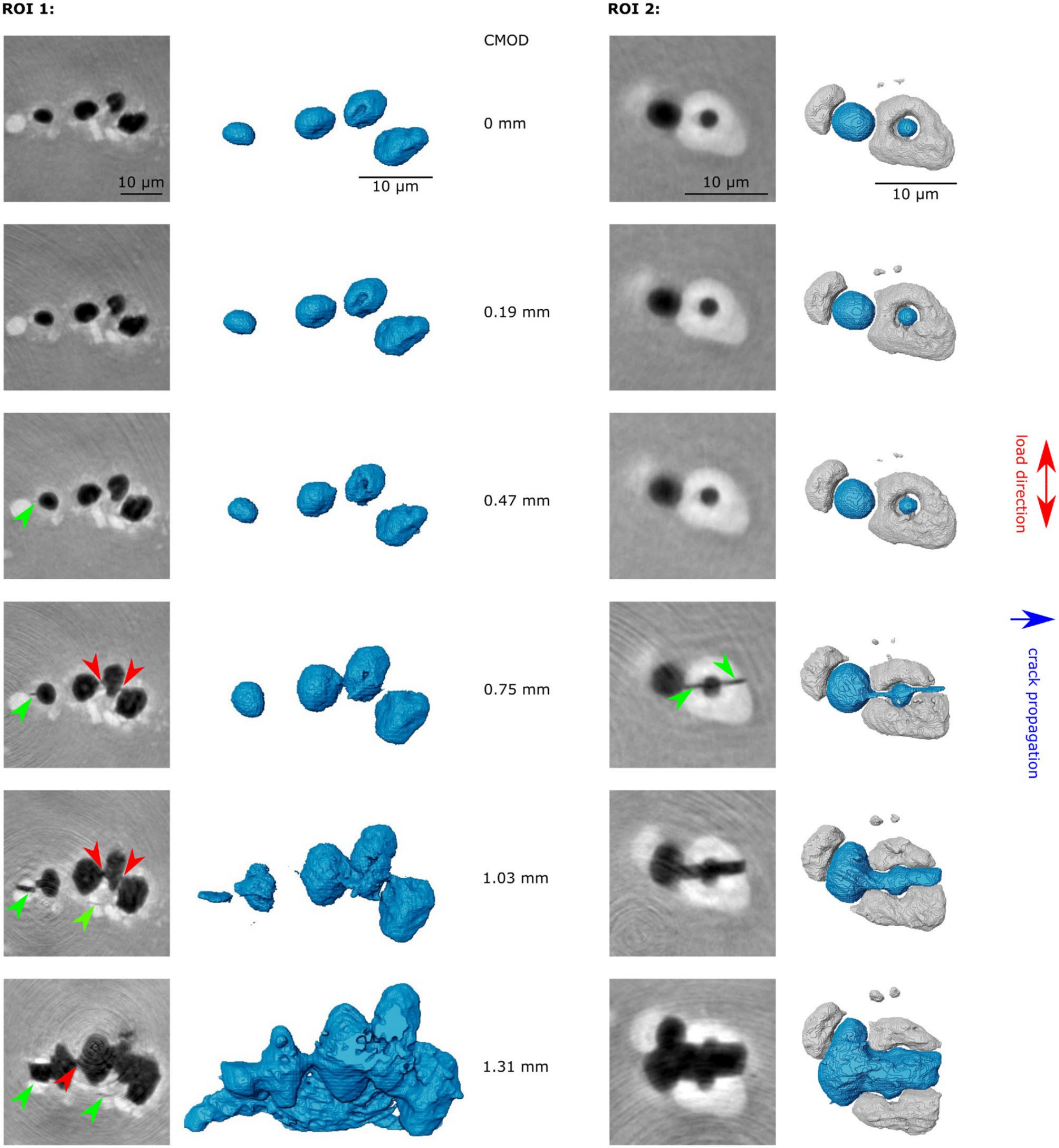
Two exemplary ROIs during in situ loading. **ROI 1**: Void cluster shown in Fig. 1d illustrating the void growth of several pores (black/blue) for increasing CMOD values within the sample. Further void nucleation occurs due to the cracking of secondary particles (green arrows). Coalescence processes of the voids (red arrows) are leading to crack propagation in the matrix. Due to the complex void-secondary phase particle configuration the segmented volume of ROI 1 only shows the voids in the material. **ROI 2:** Details of secondary phase particle cracking. The crack initiates at a pre-existing void in the secondary phase particle (white/grey) and subsequently grows with further loading. The blue arrow indicates the direction of crack propagation, the red arrow the direction of the applied force. The segmented volume shows void (blue) and secondary phase particle (grey).

**Figure 5:**
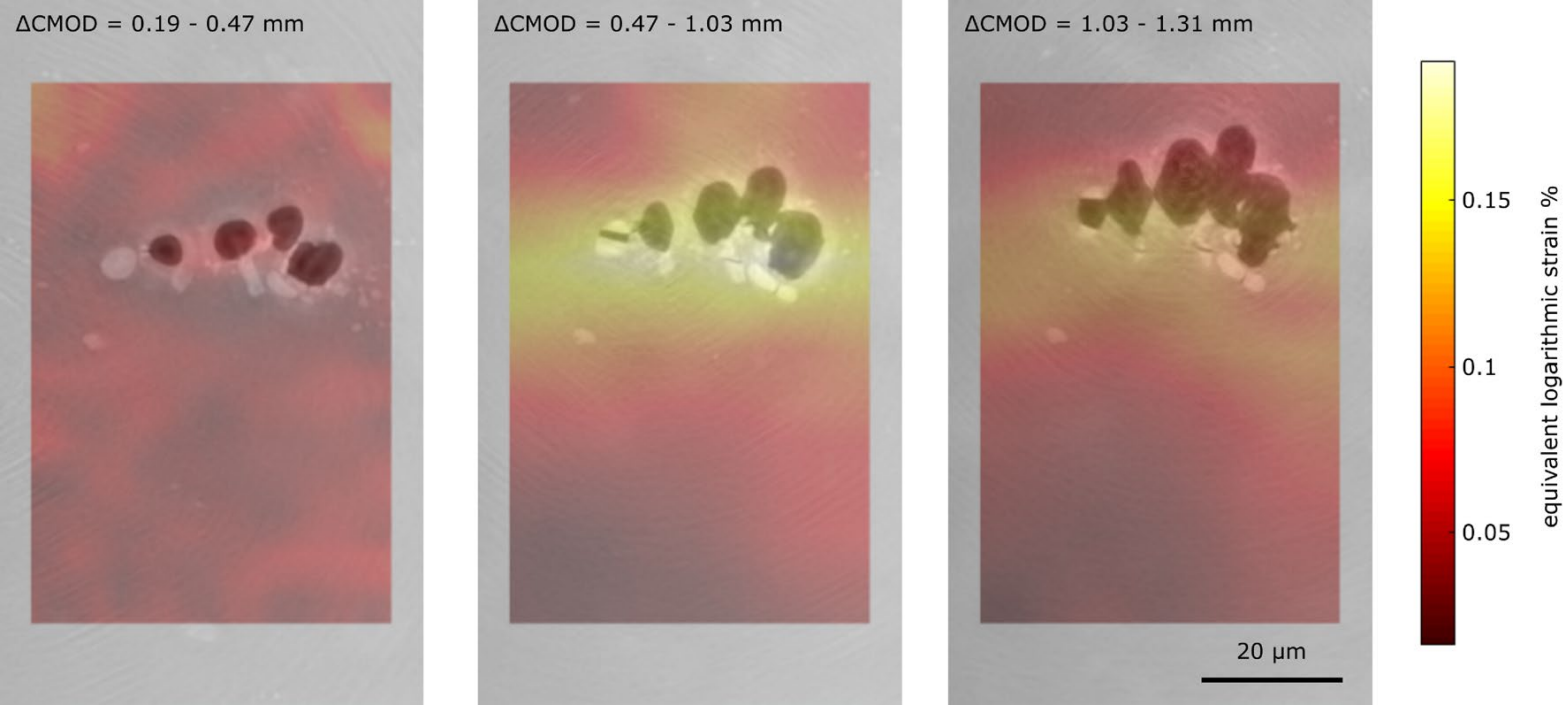
Void cluster shown in Fig. 4 with corresponding sections of equivalent, logarithmic incremental strain field as obtained by regularised 3D DVC^43^. It is clearly visible that strained bands are located around nanoscale void clusters leading to the fracture of secondary phase particles.

Figure 1d shows that the studied alloy contains three different phases i.e. voids (black), intermetallic particles (white), and the aluminium matrix (grey). By means of hierarchical guidance, clusters of voids and intermetallic particles could be identified and tracked. In Fig. 4, two examples of void clusters are depicted. The location of pre-existing voids and secondary phase particles turns out to be strongly entangled, and it is clearly visible that void growth and nucleation are strongly influenced by secondary phase inclusions. Different types of entanglement are present: e.g. secondary phase particles next to voids (ROI 1) and secondary phase particles including voids (ROI 2). Further, secondary phase particles of various shapes and sizes can be distinguished, i.e. round and fragment-like shapes with diameters from sub-micrometre up to $10{-}20~\upmu \text {m}$. By loading the sample with a high stress triaxiality, the in situ development of clusters is accessible. The main damage nucleation process can be identified as cracking of secondary phase particles. The first cracking events of secondary phase particles are observed for $\text {CMOD}=0.47\,\text {mm}$. Further particles fracture even at the last studied loading step ($\text {CMOD}=1.31\,\text {mm}$). These microstructural observations can be correlated with strain fields determined by DVC, see Fig. 5. The strain is strongly concentrated around void clusters and the strained bands formed lead to fracture of the included intermetallic particles, which nucleates new damage growing afterwards. Further, a size and shape dependence of secondary particles on their cracking behaviour is observed.

Future statistical data analyses of such morphological measures of secondary phase particles and their correlation with strain fields will allow the condition for secondary phase particle cracking to be determined as a function of strain, particle size and shape. These observations will serve as valuable input for unit cell calculations for the development of new fracture models considering the in situ microstructural development of voids and intermetallic particles, proving a comprehensive picture of damage nucleation in ductile fracture^37^.

## Conclusion

We developed the concept of hierarchically guided X-ray microscopy and presented a workflow with suitable tools to implement it. The potential of the approach for extending the capabilities of nanoscale 3D X-ray imaging was demonstrated. Instrumentally, the method exploits the zoom-capability of a projection microscopy setup, while low-latency data processing is realised by an adapted 3D reconstruction pipeline fed by a stream of on-the-fly phase-retrieved projection data. Based on this, during ongoing high-resolution experiments, a wealth of information about the current progress of the experiment can be accessed, necessary for taking decisions on the future steps as particularly crucial for many in situ measurements. In particular, this enables the identification and continuous tracking of ROIs within large samples undergoing global and local displacements as well as instant quality assurance of the measured data. We showed that, in combination with a laminographic acquisition geometry, ROIs even within objects with dimensions exceeding the field of view of the used detector by more than two orders of magnitude can be identified and selected for 3D investigation with deep sub-micrometre resolution, opening new possibilities for high-resolution in situ studies of such large samples undergoing considerable displacements or strong morphological changes.

For the realisation of the fast and high-resolution on-the-fly processing required, the adaption of non-iterative, single-distance QP phase-retrieval to cone-beam geometry was key. We presented and discussed in detail its theoretical justification, supported by a comparison of its performance with commonly used state-of-the art phase-retrieval approaches. In this way, we showed that the extended QP phase-retrieval provides similar image quality, but with sufficiently reduced time for data acquisition and processing, even for samples giving rise to considerable absorption and large phase-shifts.

Finally, we applied the method to enable the nanoscale in situ investigation of damage nucleation within the bulk of a macroscopic aluminium alloy sheet, allowing us to observe various internal crack shapes and their development during the loading progress. Moreover, the applicability of DVC to the obtained 4D nanoscale data was shown, opening new possibilities e.g. for the here presented quantitative study of strained bands located at clusters of voids and secondary phase particles. In the future, the statistical analysis of such data, in particular the correlation of nanoscale morphodynamics and the surrounding mesoscale strain states during the damage nucleation period, will pave the way towards realistic damage nucleation models accounting for secondary phase particle number, distribution, and shapes, as well as the corresponding strain states.

Beyond the application presented, X-ray projection microscopy will broadly benefit from hierarchical guidance. The approach is generally applicable and independent from many imaging parameters like tomographic or laminographic acquisition geometries or the spatial resolution. Especially at the upcoming fourth generation synchrotron sources, the presented hierarchical guidance will have the potential to boost the possibilities of nanoscale 3D imaging of  specimens of increasingly larger sizes with, at the same time, improved spatial and temporal resolutions.

## Methods

### Magnified projection microscopy

For the acquisition of scans with variable spatial resolution the slightly diverging cone-beam provided by the KB-mirror system of ID16b at the ESRF was employed (Fig. 1b). A spatial resolution down to about ${\sim 50}\,\text {nm}$ is achievable due to its reduced focal spot size^4^ and the recorded images are dominated by propagation-based phase-contrast. The KB mirrors are located ${165}\,\text {m}$ from an undulator source and provide a divergent pink beam, with an energy bandwidth $\frac{\triangle E}{E}=10^{-2}$. For the measurements presented, an X-ray energy of ${29.6}\,\text {ke}\text {V}$ was used. While Fresnel-propagation is an intrinsic property of this imaging system, phase-contrast is crucial for hierarchical guidance, since there is a trade-off between sample size, FOV, contrast and resolution; especially if high energies are necessary to penetrate large samples, phase-contrast becomes crucial for local high-resolution 3D imaging. The X-ray images have been captured by a scintillator based indirect X-ray detector system^28^, using a 10x magnification of the visible light in combination with a sCMOS camera (PCO.Edge, PCO Kelkheim, Germany). Thus, the effective pixel size of the detector was equal to ${0.65}\,\upmu \text {m}$. In combination with the geometrical magnification of the slightly diverging cone-beam, the image system provides variable resolution from ${\sim 50}\,\text {nm}$ to ${\sim 0.5}\,\upmu \text {m}$. The effective voxel size for low-resolution scans was chosen to be ${240}\,\text {nm}$ (propagation distance $d_{2} \approx 0.477~\text {m}$; X-ray magnification $M \approx 2.7$) high-resolution scans have been acquired using a voxel size of ${99.5}\,\text {nm}$ (propagation distance $d_{2} \approx$ ${0.638}\,\text {m}$; X-ray magnification $M\approx 6.5$). The low-resolution effective voxel size is chosen based on the expected shifts during the in situ experiment, the high-resolution effective voxel size is chosen based on the length scale of the relevant processes within the ROI.

### X-ray computed laminography

CL is a generalisation of CT, which allows for 3D investigation of flat and laterally extended objects. The rotation axis is inclined by the laminography angle $\theta$ with respect to the beam axis, see Fig. 1b. Here a laminographic angle of ${33}^{\circ }$ has been employed to record 3000 magnified projections from different view angles equally distributed over ${360}^{\circ }$. Similar to limited angle CT Fourier space sampling is not complete in CL^3^. However, due to the different acquisition geometry CL achieves superior image quality^7^. The specific Fourier space sampling results in a high in-plane resolution and characteristic artefacts along the rotation axis^6,38^. Laminography has been broadly applied with micrometer spatial resolution e.g.^16,17,39^. Few CL experiments at the nanoscale have been performed, all of them at energies less than ${20}\,\text {ke}\text {V}$ and with static, much smaller samples (i.e. thickness of ${40}\,\upmu \text {m}$ thickness) and (iterative) multidistance phase-retrieval^7^. Recently, scanning nanolaminography^40,41^ has been introduced at very high spatial resolution and outstanding hierarchical capabilities, but at the expense of long scan times, low energies and reduced sample thicknesses.

### Kinematic measurement: digital image and volume correlation

2D strain fields at the surface are assessed *via* DIC from a speckle pattern painted on sample surface^35^. The optical microscopy images have a pixel size of ${3.8}\, \upmu \text {m}$ and FOV of ${7.8}\,\text {mm}$. Additionally to the strain field, extensometer measurements are obtained. For 3D strain analysis regularised DVC^42^ is applied to the X-ray CL data sets. The method has been broadly applied to such materials at the micron scale and enabled internal 3D strain fields to be measured^16^. To obtain results with the sparse feature density of high-resolution scans mechanical regularisation is used^43^. Using an increased regularisation length (${25.6}\,\upmu \text {m}$) allowed low frequency, incremental strain fields to be assessed, as presented in Fig. 5, despite the low particle density within the high-resolution images. The strain fields have been verified by analysing the correlation residuals.

## Data Availability

The presented data is available from the corresponding author on reasonable request.
